# Polyamines Confer Salt Tolerance in Mung Bean (*Vigna radiata* L.) by Reducing Sodium Uptake, Improving Nutrient Homeostasis, Antioxidant Defense, and Methylglyoxal Detoxification Systems

**DOI:** 10.3389/fpls.2016.01104

**Published:** 2016-07-28

**Authors:** Kamrun Nahar, Mirza Hasanuzzaman, Anisur Rahman, Md. Mahabub Alam, Jubayer-Al Mahmud, Toshisada Suzuki, Masayuki Fujita

**Affiliations:** ^1^Laboratory of Plant Stress Responses, Faculty of Agriculture, Kagawa UniversityKagawa, Japan; ^2^Department of Agricultural Botany, Faculty of Agriculture, Sher-e-Bangla Agricultural UniversityDhaka, Bangladesh; ^3^Department of Agronomy, Faculty of Agriculture, Sher-e-Bangla Agricultural UniversityDhaka, Bangladesh; ^4^Department of Agroforestry and Environmental Science, Faculty of Agriculture, Sher-e-Bangla Agricultural UniversityDhaka, Bangladesh; ^5^Biomass Chemistry Laboratory, Bioresource Science for Manufacturing, Department of Applied Bioresource Science, Faculty of Agriculture, Kagawa UniversityKagawa, Japan

**Keywords:** abiotic stress, salinity, polyamine, methylglyoxal, oxidative damage, ROS signaling

## Abstract

The physiological roles of PAs (putrescine, spermidine, and spermine) were investigated for their ability to confer salt tolerance (200 mM NaCl, 48 h) in mung bean seedlings (*Vigna radiata* L. cv. BARI Mung-2). Salt stress resulted in Na toxicity, decreased K, Ca, Mg, and Zn contents in roots and shoots, and disrupted antioxidant defense system which caused oxidative damage as indicated by increased lipid peroxidation, H_2_O_2_ content, $O_{2}^{•-}$ generation rate, and lipoxygenase activity. Salinity-induced methylglyoxal (MG) toxicity was also clearly evident. Salinity decreased leaf chlorophyll (chl) and relative water content (RWC). Supplementation of salt affected seedlings with exogenous PAs enhanced the contents of glutathione and ascorbate, increased activities of antioxidant enzymes (dehydroascorbate reductase, glutathione reductase, catalase, and glutathione peroxidase) and glyoxalase enzyme (glyoxalase II), which reduced salt-induced oxidative stress and MG toxicity, respectively. Exogenous PAs reduced cellular Na content and maintained nutrient homeostasis and modulated endogenous PAs levels in salt affected mung bean seedlings. The overall salt tolerance was reflected through improved tissue water and chl content, and better seedling growth.

## Introduction

Plants show susceptibility to various stresses under both wild and cultivated conditions. In many areas of the world, high concentrations of salt in the soil are commonly encountered phenomenon. Salinity is a complex stress that involves both ionic and osmotic components. Salt stress adversely affects plant development and productivity by generating ion toxicity, inducing nutritional deficiencies, creating osmotic stress and water deficits, and decreasing photosynthesis (Shabala and Munns, 2012).

Reactive oxygen species are partly reduced forms of oxygen and by-products of aerobic metabolism and stronger oxidants than molecular oxygen. ROS are overproduced under stress condition and can potentially damage the intracellular machinery. ROS may include free radicals such as superoxide anion ($O_{2}^{•-}$) and hydroxyl radical (^∙^OH), as well as non-radical molecules like hydrogen peroxide (H_2_O_2_) and singlet oxygen (^1^O_2_). Plants can scavenge a certain amount of ROS generated as by-products of aerobic metabolism, but under stress conditions, including salt stress, the resulting photoinhibition often creates an imbalance between ROS generation and scavenging due to generation of excessive amount of ROS, thereby resulting in oxidative stress, causing peroxidation of lipids, oxidation of proteins, inhibition of enzyme activity, injury to nucleic acids, activation of programmed cell death (PCD) pathways, and eventually leading to death of the cells (Gill and Tuteja, 2010; Hasanuzzaman et al., 2013). The antioxidant defense machinery is a ROS scavenging system, which protects plants from oxidative damage. Efficient enzymatic (superoxide dismutase, SOD; catalase, CAT; ascorbate peroxidase, APX; glutathione reductase, GR; monodehydroascorbate reductase, MDHAR; dehydroascorbate reductase, DHAR; glutathione peroxidase, GPX; guaicol peroxidase, GOPX; and glutathione *S*-transferase, GST) and non-enzymatic (ascorbate, AsA; glutathione, GSH; phenolic compounds, alkaloids, non-protein amino acids, and α-tocopherols) components form an antioxidant defense system to protect overproduction of ROS and to prevent plant cells from experiencing oxidative damage (Gill and Tuteja, 2010).

Methylglyoxal (MG) is a transition-state intermediate product of the triose-phosphates of the glycolysis pathway in eukaryotic cells. High accumulation of MG is toxic and inhibits cell proliferation, causes degradation of proteins by modifying arginine, lycine, and cysteine residues, forms adducts with guanyl nucleotide in DNA, and inactivates antioxidant defense system, and causes oxidative stress (Yadav et al., 2005). The glyoxalase system comprises of two enzymes, gly I (glyoxalase I) and gly II (glyoxalase II), which catalyze the detoxification reaction of MG to D-lactate (Yadav et al., 2005). Although the glyoxalase enzymes have been extensively studied in microbial and animal systems (Thornalley, 1990), the biological significance of this pathway in plants and under stress condition is just beginning to be explored (Yadav et al., 2005).

The polyamines (PAs) are low-molecular-weight organic cations found in a wide range of organisms, where they perform diverse biological functions (Kusano et al., 2008). Putrescine (Put), spermidine (Spd), and spermine (Spm) are the most common PAs. The levels of PAs frequently increase during stress and they play roles in enhancing plant stress tolerance. Acting as molecular chaperones, PAs bind to negatively charged surfaces and protect membranes and biomolecules (Kusano et al., 2008). PAs may also act as ROS and free-radical scavengers and activate the antioxidant enzyme machinery which help to reduce oxidative stress and subsequent membrane injury and electrolyte leakage (Gill and Tuteja, 2010; Kubiś et al., 2014). PAs interact with some signaling molecules (Kusano et al., 2008). PAs have been confirmed to be unique polycationic metabolites involved in the direct blockage of a variety of cation and K^+^-selective channels, vacuolar-type channels, and ammonium channels (Dobrovinskaya et al., 1999). PAs restrict plasma membrane Na^+^ influx and NaCl-induced K^+^ efflux and shoot-to-root K^+^ recirculation (Shabala et al., 2007; Zhao et al., 2007). Despite their range of protective effects under stress condition, acute application of PAs can cause endogenous PAs catabolism in the apoplast, which is responsible for ROS-induced oxidative damage (Di Tomaso et al., 1989). The diversity of the physiological functions of PAs has made understanding the detailed mechanisms of the protective function of PAs in response to stress particularly challenging.

Salt stress tolerance is a polygenic trait. Some of the major traits affected by salinity include the maintenance of membrane transport activity and ionic homeostasis, compartmentalization of ions at the cellular and whole-plant level (which may include Na^+^ exclusion from root uptake, intra-cellular Na^+^ sequestration, cytosolic K^+^ retention), osmotic adjustment through synthesis of compatible solutes, oxidative stress tolerance, biomembrane protection, and MG toxicity tolerance (Yadav et al., 2005; Shabala and Munns, 2012; Rahman et al., 2016). Changes in ranges of biochemical pathways and the induction of hormones and signaling molecules activate stress responsive genes that impart salt stress tolerance (Adem et al., 2014). Polyamines have diverse mechanism to enhance salt tolerance. Under salt stress PAs proved to be efficient in modulating membrane integrity and ion transport processes, vacuolar ion channels, shoot, and root growth (Di Tomaso et al., 1989; Dobrovinskaya et al., 1999), photosynthesis (Kotzabasis et al., 1993; Duan et al., 2008; Shu et al., 2012), antioxidative responses (Sheokand et al., 2008; Ikbal et al., 2014); Na^+^ and nutrient influx/efflux (Shabala et al., 2007; Pottosin et al., 2012). However, the actual mechanism by which PAs modulate these physiological processes is not yet known. A few components of antioxidant system were studied in response to PAs modulation. Polyamine-induced MG detoxification process is unknown in plants. In the present study, we have investigated the roles of PAs in maintaining ionic and nutrient homeostasis, inducing osmotic adjustment, and protecting mung bean plant cells from oxidative damage and MG toxicity under salt stress. The results presented here provide new insight into the role of PAs in enhancing salt tolerance and mitigating salt stress-induced damage in mung bean seedlings by the coordinated action of PAs on ion homeostasis, antioxidant defense, and the glyoxalase system.

## Materials and Methods

### Plant Materials, Growth Condition, and Stress Treatments

Mung bean (*Vigna radiata* L. cv. BARI Mung-2) seedlings were grown in petri dishes under the conditions: light, 350 μmol photon m^-2^ s^-1^; temperature, 25 ± 2°C; relative humidity, 65–70%; 10,000-fold diluted Hyponex solution (Hyponex, Japan) was applied as nutrient. Six-day-old seedlings (three sets) were exposed to salt stress (NaCl, 200 mM). Three sets of 5-day-old seedlings were grown with Put (0.2 mM), Spd (0.2 mM), and Spm (0.2 mM) solution as pre-treatment for 24 h. These pre-treated seedlings were then exposed to the same level of salt stress on day six. Control seedlings were grown with Hyponex solution. Another three sets of seedlings were grown with Put, Spd and Spm without any stress. Data were taken after 48 h. There were three replicates per treatment.

### Measurement of Na and Mineral Nutrients Contents in Roots and Shoots

Root and shoot samples were oven-dried at 80°C for 48 h. Dried samples were ground and subjected to acid digestion in HNO_3_:HClO_4_ (5:1 v/v) mixture at 80°C. The Na, K, Ca, Mg, and Zn contents were measured using flame atomic absorption spectrophotometer.

### Histochemical Detection of Hydrogen Peroxide and Superoxide

The H$O_{2}^{•-}$ and $O_{2}^{•-}$ were localized histochemically (Chen et al., 2010) by staining leaves with 1% 3,3-diaminobenzidine (DAB) and 0.1% nitroblue tetrazolium chloride (NBT) solution, respectively.

### Lipid Peroxidation, H_2_O_2_ Content and $O_{2}^{•-}$ Generation Rate

The level of lipid peroxidation was measured by estimating (MDA, a product of lipid peroxidation) using TBA according to Heath and Packer (1968) with modifications (Hasanuzzaman et al., 2011). Hydrogen peroxide (H_2_O_2_) was assayed according to Yu et al. (2003) by extracting leaves in potassium-phosphate buffer (pH 6.5) (centrifuging at 11,500 × *g*), then adding it to a mixture of TiCl_4_ in 20% H_2_SO_4_ (v/v) and the resulting solution was measured spectrophotometrically at 410 nm. To measure rate of $O_{2}^{•-}$ generation leaves were homogenized in K–P buffer solution, centrifuged at 5000 × *g*. Supernatant was mixed with extraction buffer and hydroxylamine hydrochloride for incubation; then mixed with sulfanilamide and naphthylamine, incubated at 25°C. The absorbance was measured at 530 nm (Nahar et al., 2016).

### Extraction and Measurement of Ascorbate and Glutathione

Leaves (0.5 g) were homogenized in 5% meta-phosphoric acid containing 1 mM EDTA (centrifuged at 11,500 × *g*) for 15 min at 4°C and the supernatant was collected for analysis of ascorbate and glutathione. Ascorbate content was determined following the method of Hasanuzzaman et al. (2011). To determine total ascorbate, the oxidized fraction was reduced by adding 0.1 M dithiothreitol for 1 h at room temperature and then read at 265 nm using 1.0 unit AO. Oxidized ascorbate (DHA) content was determined by subtracting reduced AsA from total AsA. The glutathione pool was assayed according to previously described methods (Yu et al., 2003) with modifications as described by Hasanuzzaman et al. (2011). Standard curves with known concentrations of GSH and GSSG were used. The content of GSH was calculated by subtracting GSSG from total GSH.

### Protein Determination and Enzyme Extraction and Assays

The protein concentration was determined according to Bradford (1976). Leaves were homogenized with 50 mM K–P (potassium phosphate) buffer (pH 7.0) containing 100 mM KCl, 1 mM AsA, 5 mM β-mercaptoethanol, and 10% (w/v) glycerol, centrifuged at 11,500 × *g* and supernatants were used for enzyme activity assay. SOD (EC 1.15.1.1) activity (El-Shabrawi et al., 2010), CAT (EC: 1.11.1.6) activity (Hasanuzzaman et al., 2011), APX (EC: 1.11.1.11) (Nakano and Asada, 1981), MDHAR (EC: 1.6.5.4) (Hossain et al., 1984), DHAR (EC: 1.8.5.1) (Nakano and Asada, 1981), GR (EC: 1.6.4.2) (Hasanuzzaman et al., 2011), and GST (EC: 2.5.1.18) (Hossain et al., 2006) activities were measured following standard methodologies. GPX (EC: 1.11.1.9), Gly I (EC: 4.4.1.5), Gly II (EC: 3.1.2.6) activity was measured following Hasanuzzaman et al. (2011). LOX (EC 1.13.11.12) activity was estimated as described in Nahar et al. (2016).

### Methylglyoxal Level

Leaves were homogenized in 5% perchloric acid and centrifuged at 11,000 × *g*, supernatant was decolorized, neutralized, MG content was estimation by adding sodium dihydrogen phosphate and *N*-acetyl-L-cysteine to a final volume of 1 mL. Formation of the product N-α-acetyl-*S*-(1-hydroxy-2-oxo-prop-1-yl)cysteine was recorded after 10 min at a wavelength of 288 nm (Wild et al., 2012).

### Measurement of Free Polyamine Content

Endogenous free PAs were estimated according to Kotzabasis et al. (1993). In brief, leaf tissue (0.1 g) was homogenized in 1 mL of 5% (v/v) cold perchloric acid (PCA). The homogenates were kept at 2°C for 2 h and centrifuged at 15,000 × *g* for 20 min. The supernatant was collected and stored at 2°C. PA standards, free PAs were benzoylated. Briefly, 1 ml of 2N NaOH and 10 μl of benzoylchloride were added to 200 μl of the PA aliquots and vortexed for 30 s. After 20 min incubation at 25°C, 2 ml of saturated NaCl was added to stop the reaction. The benzoyl – PAs were gently mixed with 2 ml diethyl ether. After properly vortex, upper portion was taken and the ether phase was collected and evaporated to dryness in a water bath (60°C). The benzoyl-PAs were redissolved in 200 μl of 64% (v/v) methanol and analyzed by HPLC. A 20 μl of the benzoylated extract was injected into the C18 reverse phase HPLC column (4.6 mm × 100 mm, 5 μm particle size, Inertsil.ODS-3). The mobile phase was methanol and water in the ratio of 64:36, respectively, at a isocratic flow rate of 1.0 ml min^-1^ and peaks were detected with a UV detector at 254 nm. Three polyamine standards of Put, Spd and Spm were prepared at different concentrations for the production of standard curves. The final PAs content was expressed as μmol g^-1^ DW.

### Leaf Relative Water Content

Leaf relative water content (RWC) of leaf was measured according to Barrs and Weatherley (1962). Fresh weight (FW), turgid weight (TW), and dry weight (DW) of leaves were measured, and RWC was calculated using the following formula: RWC (%) = [(FW–DW)/(TW–DW)] × 100.

### Proline Content

Proline (Pro) was calculated according to Bates et al. (1973). Leaves were homogenized in 3% sulphosalicyclic acid and centrifuged at 11,500 × *g*. Supernatant was mixed with acid ninhydrin with glacial acetic acid and phosphoric acid. After incubating the mixture at 100°C for 1 h and cooling, toluene was added; chromophore containing toluene was read spectrophotometrically at 520 nm.

### Chlorophyll Content

Leaf supernatant was extracted with 80% v/v acetone (centrifuging at 5,000× *g*), absorbances were taken with a UV-visible spectrophotometer at 663 and 645 nm for chl *a* and chl *b*, respectively, and chl content was calculated according to Arnon (1949).

### Determination of Growth Parameters

Plant height and root length were measured from each set of seedlings. Ten randomly selected fresh seedlings from each treatment were dried at 80°C for 48 h, then weighed and considered as DW.

### Statistical Analysis

All data obtained were subjected to analysis of variance (ANOVA) and the mean differences were compared by Tukey’s HSD (honest significant difference) test using XLSTAT v. 2015.1.01 software (Addinsoft, 2015). Differences at *P* ≤ 0.05 were considered significant.

## Results

### Exogenous PAs Reduces Na Uptake

**FIGURE 1:**
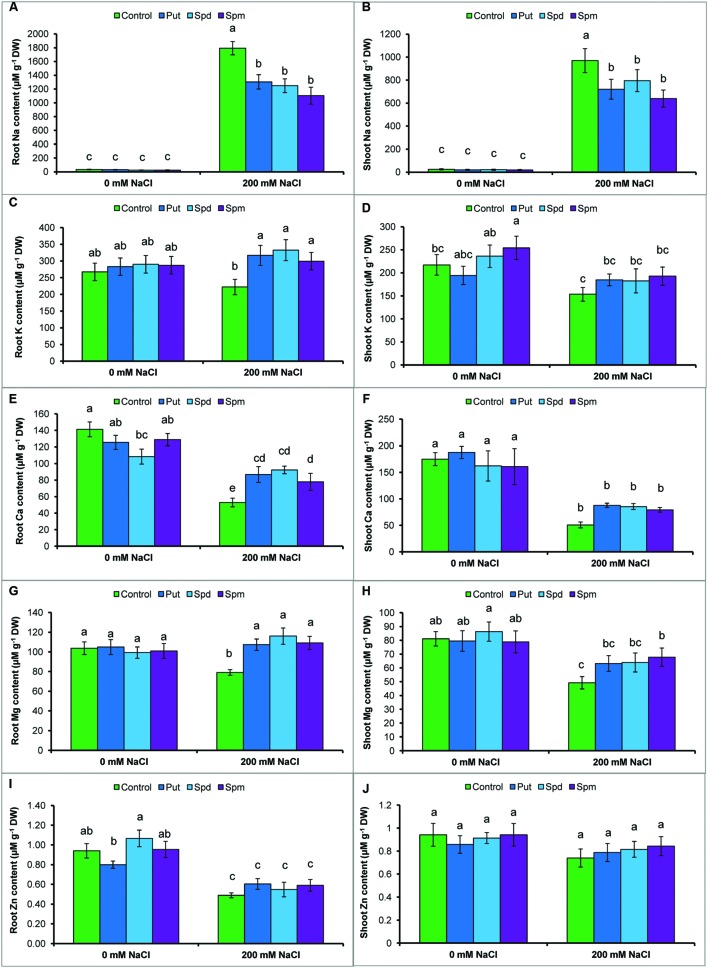
**Effect of exogenous polyamines on Na uptake and nutrient homeostasis in mung bean seedlings under salt stress (NaCl, 200 mM).** Root Na **(A)**, shoot Na **(B)**, root K **(C)**, and shoot K **(D)** root Ca **(E)**, shoot Ca **(F)**, root Mg **(G)**, shoot Mg **(H)**, root Zn **(I)**, and shoot Zn **(J)** contents. Here, Put, Spd, and Spm indicate putrescine (0.2 mM), spermidine (0.2 mM), and spermine (0.2 mM), respectively. Mean (±SD) was calculated from three replicates for each treatment. Bars with different letters are significantly different at *P* ≤ 0.05 applying Tukey’s HSD test.

The protective role of PAs against salt stress was examined by determining the Na content in roots and shoots of the mung bean seedlings. As shown in **Figures 1A,B**, salt treatment resulted in a marked increase in Na contents in the roots and shoots when compared to control seedlings. However, the application of exogenous PAs significantly decreased Na level in root and shoot (**Figures 1A,B**).

### Mineral Nutrient Contents Induced by Exogenous PAs under Salt Stress

The levels of K in the roots and shoots, decreased significantly under NaCl stress. However, the application of exogenous PAs significantly increased K level in the roots and shoots exposed to salt stress, in contrast to salt treatment without PAs (**Figures 1C,D**). Root and shoot Ca content decreased by 63 and 71%, respectively; Mg content by 24 and 39%, respectively; and Zn content by 48 and 21%, respectively in salt affected mung bean seedlings when compared to control seedlings. Exogenous supplementation of PAs together with the salt treatment increased Ca content in root and Mg content in roots and shoots, compared to the salt treatment alone. Polyamine application with salt stress slightly increased the Zn level in roots and shoots, but the difference was not statistically significant (**Figures 1E–J**).

### Histochemical Detection of ROS

**FIGURE 2:**
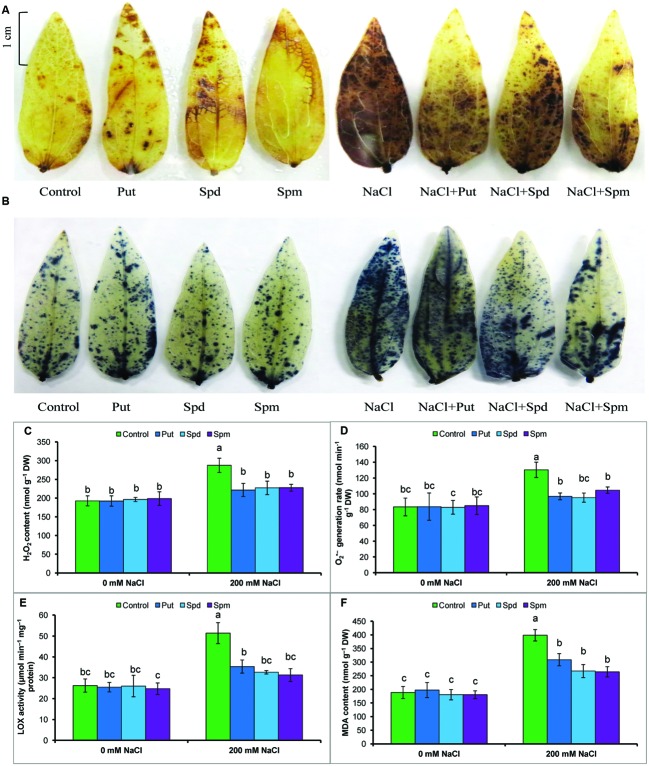
**Histochemical detection of ROS in leaves, content of ROS, LOX activity, and membrane lipid peroxidation in salt (NaCl, 200 mM) affected mung bean seedlings.** DAB staining **(A)** of H_2_O_2_ and NBT staining **(B)** of $O_{2}^{•-}$ in leaves, H_2_O_2_ content **(C)**, $O_{2}^{•-}$ generation rate **(D)**, LOX activity **(E)**, and MDA content **(F)**. Here, Put, Spd, and Spm indicate putrescine (0.2 mM), spermidine (0.2 mM), and spermine (0.2 mM), respectively. Mean (±SD) was calculated from three replicates for each treatment. Bars with different letters are significantly different at *P* ≤ 0.05 applying Tukey’s HSD test.

The accumulations of the ROS, H_2_O_2_, and $O_{2}^{•-}$, were detected by histochemical staining with DAB or NBT, respectively. Both H_2_O_2_ and $O_{2}^{•-}$ staining is clearly observed in leaves as brown patches and dark blue spots, respectively. Leaves of salt-treated mung bean seedlings had more prominent and frequent spots. Exogenous PAs reduced the numbers of spots due to H_2_O_2_ and $O_{2}^{•-}$ in the leaves of salt-treated plants (**Figures 2A,B**).

### Reactive Oxygen Species and Oxidative Stress Marker

Salt stress results in a ROS burst in mung bean seedlings. Thus, compared to control, high increases in H_2_O_2_ content and $O_{2}^{•-}$ generation rate were evident in salt-affected seedlings. The increase in MDA/lipid peroxidation, indicating damage to membranes, paralleled the accumulation of ROS (H_2_O_2_ and $O_{2}^{•-}$) and the increased activity of lipid degrading enzymes/LOX (**Figures 2C–F**).

Exogenous PAs reduced ROS accumulation in the leaves. Put, Spd, and Spm supplementation during salt stress reduced H_2_O_2_ content by 23, 21, and 21%, respectively, and reduced $O_{2}^{•-}$ generation rate by 27, 27, and 20%, respectively (compared to salt affected seedlings without PA application). LOX activity was also decreased by PAs. Lipid peroxidation or MDA content was reduced by 26, 35, and 39% after Put, Spd, and Spm supplementation of salt-stressed plants (compared to salt treatment alone) (**Figures 2C–F**).

### Non-enzymatic Antioxidants

**FIGURE 3:**
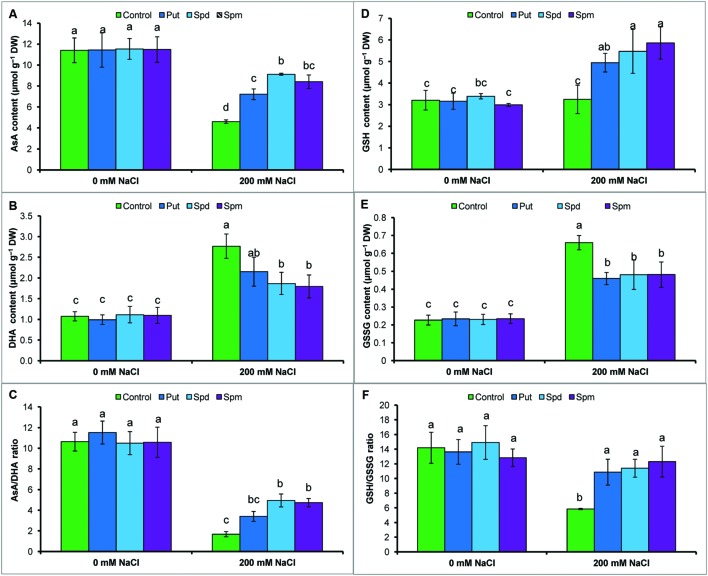
**Effect of exogenous polyamines on non-enzymatic antioxidants in mung bean seedlings under salt (NaCl, 200 mM) stress.** Ascorbate (AsA) content **(A)**, DHA content **(B)**, AsA/DHA ratio **(C)**, GSH content **(D)**, GSSG content **(E),** and GSH/GSSG ratio **(F)**. Here, Put, Spd, and Spm indicate putrescine (0.2 mM), spermidine (0.2 mM), and spermine (0.2 mM), respectively. Mean (±SD) was calculated from three replicates for each treatment. Bars with different letters are significantly different at *P* ≤ 0.05 applying Tukey’s HSD test.

Ascorbate content reduced under salt stress, whereas DHA content increased. Salt stress resulted in a decrease in AsA/DHA ratio (**Figures 3A–C**). Glutathione and GSSG contents increased, but the ratio of GSH/GSSG decreased in response to salt stress, when compared to the control seedlings (**Figures 3D–F**). Exogenous application of Put, Spd, and Spm decreased DHA content and increased content of AsA and the ratio of AsA/DHA (**Figures 3A–C**). Application of PAs also increased GSH content and decreased GSSG content, thereby increasing the ratio of GSH/GSSG (**Figures 3D–F**).

### Antioxidants Enzymes

The activity of SOD increased by 49% in plants under salt stress compared to control seedlings. Exogenous PAs application to the salt affected seedlings did not increase SOD activity further but maintained the same activity as seen in salt affected seedlings (**Table 1**). CAT activity decreased by 50% due to salt stress compared to control seedlings. Exogenous Put, Spd, and Spm restored and increased CAT activity in salt stressed mung bean seedlings (compared to seedlings treated with salt only) (**Table 1**).

**Table 1:** Effect of exogenous polyamines on antioxidant enzymes in salt (200 mM NaCl) affected mung bean seedlings.

Treatments	SOD activity (U min^-1^ mg^-1^ protein)	CAT ctivity (μmol min^-1^ mg^-1^ protein)	APX activity (μmol min^-1^ mg^-1^ protein)	MDHAR activity (nmol min^-1^ mg^-1^ protein)	DHAR activity (nmol min^-1^ mg^-1^ protein)	GR activity (nmol min^-1^ mg^-1^ protein)	GPX activity (nmol min^-1^ mg^-1^ protein)	GST activity (nmol min^-1^ mg^-1^ protein)
Control	72 ± 11b	84 ± 7ab	0.557 ± 0.05abc	35 ± 1.1a	79 ± 11ab	25 ± 2d	0.038 ± 0.01b	9.5 ± 1b
Put	69 ± 5b	91 ± 9a	0.569 ± 0.07abc	37 ± 0.1a	87 ± 10a	26 ± 3d	0.035 ± 0.01b	10.3 ± 1b
Spd	73 ± 10b	87 ± 11a	0.523 ± 0.06bc	33 ± 3.6ab	82 ± 12a	26 ± 2d	0.036 ± 0.01b	10.0 ± 2b
Spm	73 ± 2b	87 ± 12a	0.479 ± 0.06c	32 ± 2.0abc	80 ± 13ab	25 ± 3d	0.036 ± 0.01b	9.9 ± 1b
NaCl	108 ± 7a	40 ± 6c	0.703 ± 0.08ab	25 ± 2.6d	42 ± 6c	33 ± 4bcd	0.043 ± 0.01b	17.9 ± 2a
NaCl+Put	121 ± 17a	59 ± 9bc	0.723 ± 0.1a	28 ± 2.3bcd	58 ± 1bc	42 ± 4bc	0.078 ± 0.01a	16.1 ± 1a
NaCl+Spd	126 ± 8a	75 ± 11ab	0.698 ± 0.04ab	26 ± 2.9cd	70 ± 0.5ab	53 ± 5a	0.078 ± 0.02a	16.8 ± 2a
NaCl+Spm	120 ± 11a	67 ± 10ab	0.695 ± 0.05ab	28 ± 1.3bcd	64 ± 0.8abc	46 ± 3ab	0.077 ± 0.01a	15.9 ± 0.2a

*Activities of SOD (U min^-1^ mg^-1^ protein), CAT (μmol min^-1^ mg^-1^ protein), APX (μmol min^-1^ mg^-1^ protein), MDHAR (nmol min^-1^ mg^-1^ protein), DHAR (nmol min^-1^ mg^-1^ protein), GR (nmol min^-1^ mg^-1^ protein), GPX (nmol min^-1^ mg^-1^ protein), and GST (nmol min^-1^ mg^-1^ protein). Put indicates 0.2 mM putrescine, Spd indicates 0.2 mM spermidine, and Spm indicates 0.2 mM spermine. Mean (±SD) was calculated from three replicates for each treatment. Values with different letters are significantly different at P ≤ 0.05 applying Tukey’s HSD test.*

The activity of APX increased under salt stress (compared to control). In contrast to the control, salt affected mung bean seedlings showed reductions in MDHAR and DHAR activities. GR activity remained unchanged in salt stressed seedlings, compared to control plants. Activities of APX and MDHAR did not show further increases in response to the application of exogenous PAs with salt stress (when compared to plants treated with salt only). Exogenous PAs application with salt stress enhanced activities of DHAR and GR, when compared to plants exposed to the salt treatment alone (**Table 1**).

The activity of GST increased by 88% under salt stress compared to control plants. When compared to salt stress alone, the addition of PAs to salt stressed plants resulted in the same GST activity (**Table 1**). Salt stress did not significantly affect the GPX activity, when compared to control plants. In contrast, salt stressed seedlings supplemented with PAs showed higher GPX activity, when compared to plants treated with salt alone (**Table 1**).

### Methylglyoxal Toxicity and Glyoxalase System

**FIGURE 4:**
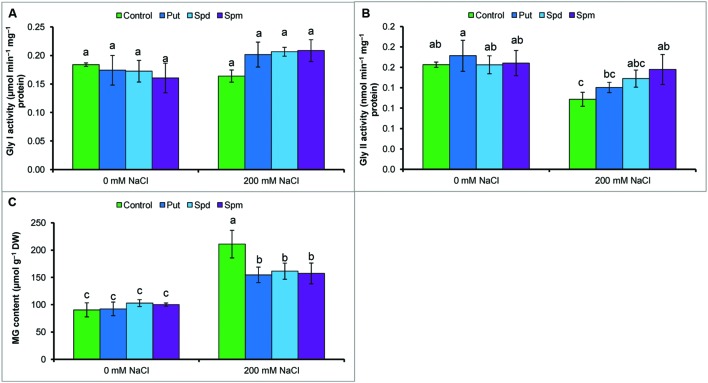
**Effect of exogenous polyamines on glyoxalase enzymes and in reducing MG toxicity in mung bean seedlings subjected to salt (NaCl, 200 mM) stress.** Activity of Gly I **(A)**, activity of Gly II **(B)**, and MG content **(C)**. Here, Put, Spd, and Spm indicate putrescine (0.2 mM), spermidine (0.2 mM), and spermine (0.2 mM), respectively. Mean (±SD) was calculated from three replicates for each treatment. Bars with different letters are significantly different at *P* ≤ 0.05 applying Tukey’s HSD test.

Methylglyoxal content increased by 109% under salt stress compared to control. Activity of Gly I remained unchanged but Gly II reduced by 33% in salt treated seedlings. However, salt affected seedlings supplemented with Put, Spd, and Spm showed enhanced Gly II activity and reduced MG contents (compared to salt stressed seedlings without any exogenous protectant) (**Figures 4A–C**).

### Endogenous Free Polyamines Contents

**FIGURE 5:**
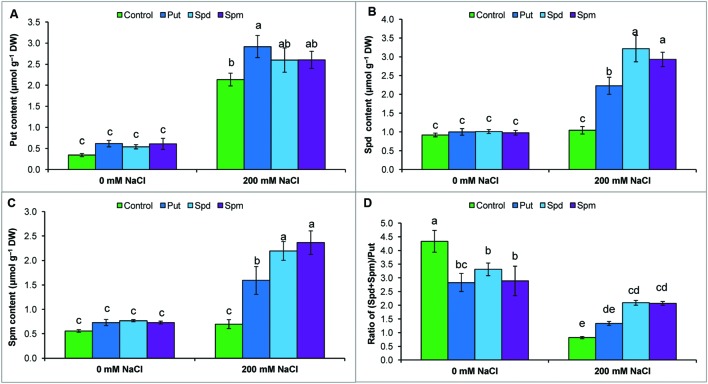
**Endogenous levels of free polyamines in salt (NaCl, 200 mM) affected mung bean seedlings induced by treatment with exogenous polyamines.** Endogenous Put **(A)**, Spd **(B)**, and Spm **(C)** contents and the ratio of (Spd+Spm)/Put **(D)**. Here, Put, Spd, and Spm indicate exogenous putrescine (0.2 mM), spermidine (0.2 mM), and spermine (0.2 mM), respectively. Mean (±SD) was calculated from three replicates for each treatment. Bars with different letters are significantly different at *P* ≤ 0.05, applying Tukey’s HSD test.

Mung bean seedlings exposed to salt stress accumulated high levels of endogenous free Put, Spd, and Spm (compared to control). The highest increase was observed for Put content, which reduced the ratio of (Spd+Spm)/Put. Exogenous application of PAs to salt stressed seedlings did not change the Put content, but increased Spd and Spm contents, which restored and enhanced the ratio of (Spd+Spm)/Put (**Figures 5A–D**).

### Leaf RWC and Pro Content

Tissue dehydration was a characteristic symptom of salt affected seedlings and was exhibited as a reduction in leaf RWC, when compared to control plants. Increases in the content of the cellular osmolyte Pro were associated with reduced RWC. Exogenous Put, Spd, and Spm addition to salt-stressed plants resulted in maintenance of the same level of Pro as seen in the salt-affected seedlings, but increased the leaf RWC (**Table 2**).

**Table 2:** Leaf (RWC, %), (Pro, μmol g^-1^ DW) content, chl *a*, chl *b*, and chl (*a*+*b*) contents (mg g^-1^ DW), plant height (cm), root length (cm), and DW (g seedling^-1^) of salt (200 mM NaCl) affected mung bean seedlings induced by exogenous polyamines.

Treatments	RWC (%)	Pro content (μmol g^-1^ DW)	chl *a* (mg g^-1^ DW)	chl *b* (mg g^-1^ DW)	Chl (*a*+*b*) (mg g^-1^ DW)	Plant height (cm)	Root length (cm)	Dry weight (g seedling^-1^)
Control	91 ± 0.8a	22.74 ± 1.25b	17.12 ± 1.89a	7.86 ± 1.20ab	24.97 ± 0.77a	9.5 ± 0.7a	9.2 ± 0.8a	0.024 ± 0.003a
Put	90 ± 1.5a	24.89 ± 4.25b	17.23 ± 2.11a	8.06 ± 1.41a	25.28 ± 3.32a	9.3 ± 0.8a	9.2 ± 0.9a	0.025 ± 0.002ab
Spd	92 ± 1.8a	25.15 ± 3.54b	17.47 ± 1.32a	7.31 ± 0.51ab	24.77 ± 1.54a	9.6 ± 0.6a	9.3 ± 0.7a	0.025 ± 0.003ab
Spm	93 ± 1.8a	25.55 ± 2.78b	17.13 ± 1.78a	7.43 ± 1.13ab	24.55 ± 1.21a	9.7 ± .9a	9.3 ± 0.9a	0.026 ± 0.003a
NaCl	70 ± 0.6c	52.03 ± 2.76a	6.60 ± 0.31c	4.98 ± 0.41b	11.57 ± 0.52c	7.9 ± 0.6b	7.1 ± 0.7b	0.017 ± 0.001b
NaCl+Put	85 ± 0.9b	53.55 ± 5.33a	10.18 ± 1.84bc	6.96 ± 1.05ab	17.13 ± 2.29b	8.7 ± 0.9ab	8.1 ± 0.8ab	0.022 ± 0.003ab
NaCl+Spd	84 ± 2.7b	58.93 ± 5.54a	10.74 ± 0.92bc	7.03 ± 1.39ab	17.76 ± 0.48b	8.6 ± 0.7ab	8.2 ± 0.7ab	0.021 ± 0.002ab
NaCl+Spm	84 ± 1.6b	55.62 ± 7.77a	11.19 ± 1.32c	6.68 ± 0.75ab	17.87 ± 0.66b	8.7 ± 0.9ab	8.3 ± 0.9ab	0.023 ± 0.002ab

*Put indicates 0.2 mM putrescine, Spd indicates 0.2 mM spermidine, and Spm indicates 0.2 mM spermine. Mean (±SD) was calculated from three replicates for each treatment. Values with different letters are significantly different at P ≤ 0.05 applying Tukey’s HSD test.*

### Photosynthetic Pigments

Salt stress treatment decreased the contents of photosynthetic pigments. Reductions in chl *a*, chl *b*, and total chl (*a*+*b*) contents under salt stress were 61, 36, and 54%, respectively. Exogenous PAs restored and increased content of chl (**Table 2**).

### Seedling Growth and Development

Salt stress inhibited the growth of mung bean seedlings. A 17% reduction in plant height was noticed in salt affected mung bean seedlings, when compared to control seedlings. Root length of salt affected seedlings reduced by 23%, compared to control seedlings without salt stress. Reduction in these growth parameters resulted in a significant reduction in seedling DW. Exogenous Put, Spd, and Spm alleviated the adverse affects of salt stress and improved growth of seedlings, as indicated by the increased plant height, root length, and seedling DW (when compared to salt treatment alone) (**Table 2**).

## Discussion

Polyamines play multifunctional roles to improve plant physiology both under normal growth condition and under stress condition. Reduction of Na influx, modulation of ion transport processes (Di Tomaso et al., 1989; Dobrovinskaya et al., 1999), upregulation of antioxidant system and alleviation of oxidative stress, osmotic adjustment (Sheokand et al., 2008; Ikbal et al., 2014) and improvement of photosynthesis (Kotzabasis et al., 1993; Duan et al., 2008; Shu et al., 2012) are some vital functions of PAs improving salt tolerance in plants. However, in previously conducted experiment some physiological parameters were studied but the actual mechanism by which PAs modulates these physiological processes were not executed. A few components of antioxidant system were studied under salt stress in response to PAs. Polyamine-induced MG detoxification process is unexplained in plants. Present study mainly focuses on the antioxidant system and oxidative stress in detail. We execute the MG toxicity under salt stress and find out the roles of PAs to alleviate MG toxicity. We also investigate roles of PAs in reducing Na uptake, maintaining nutrient homeostasis, osmotic adjustment, plant water status, protecting photosynthetic pigments from salt damage and overall growth performance of mung bean seedlings.

An influx of Na^+^ into root epidermal cells through plasma membrane non-selective channels, followed by a depolarization and activation of the K^+^ efflux, are the immediate and primary responses of plants exposed to salt stress (Shabala et al., 2007; Zhao et al., 2007). Salt-affected mung bean seedlings showed a higher Na accumulation and reduced K level, (**Figures 1A–D**). Replacement of Na^+^ by K^+^, Na^+^ exclusion, and retention of intracellular K^+^ are all important salt tolerance mechanisms (Shabala et al., 2007). An increase in K and decrease in Na level in PAs-supplemented salt-affected seedlings (**Figures 1A–D**) indicated the influential roles of PAs in reducing Na toxicity. Zhao et al. (2007) revealed that PAs reduced Na uptake, increased K uptake and improved K^+^/Na^+^ homeostasis by regulating root ion channel activities, thereby enhancing salt tolerance in barley seedlings. Exogenously applied PAs inhibited non-selective cation channels and decreased NaCl-induced membrane depolarization and K^+^ efflux in pea mesophyll cells (Shabala et al., 2007).

Imbalances in nutrient homeostasis were another component of the salt-induced damage in mung bean seedlings. Depolarization of root plasma membrane, disruption of plasma membrane ion channels, replacement of nutrient by excess Na influx are reasons for reduction of nutrient uptake and imbalances in nutrient homeostasis under salt stress (Shabala et al., 2007; Zhao et al., 2007). Reductions in Ca, Mg, and Zn content in roots and shoots were clearly evident in the salt-stressed seedlings (**Figures 1E–J**). However, exogenous Put, Spd, or Spm alleviated the negative effects of salt stress by increasing the mineral nutrient levels (except for Zn content), when compared to salt stress only (**Figures 1E–J**). Pottosin et al. (2012) reported that PAs improve membrane transportation, regulate movement of cation/anion through membrane and maintained nutrient homeostasis. Further clarification of PAs-induced mineral homeostasis will add value to this research topic.

Under salt stress, imposition of ionic and osmotic stress interrupts the soil-plant water relationship and reduces plant water uptake, thereby causing physiological drought (Shabala and Munns, 2012). Salt stress-induced osmotic stress is one of the primary detrimental effects that induce expression of compatible solute associated genes, *de novo* synthesis of compatible solutes (Shabala and Munns, 2012; Adem et al., 2014). Similar physiological drought and osmotic stress was distinguished in salt affected mung bean seedlings as indicated by reduced leaf RWC and accumulation of Pro. Exogenous PAs application to salt-stressed plants maintained the Pro level at the same level as seen in the salt only treatment, but increased the leaf RWC (**Table 2**). Here, together with Pro, PAs were supposed to be involved in the regulation of other osmoprotectant molecules that maintained the tissue water content/leaf RWC at a high level (Duan et al., 2008; Adem et al., 2014; Kubiś et al., 2014).

Salt increases the activity of the chlorophyllase enzyme, making the pigment–protein complex unstable, while salt-induced ROS degrade photosynthetic pigments (Shu et al., 2012). Salt stress decreased leaf photosynthetic pigments, including chl *a* and total chl (*a*+*b*) (compared to control) and exogenous PAs supplementation to salt-stressed plants improved chl (*a*+*b*) content (**Table 2**). PAs rapidly enter the chloroplast, where they protect the photosynthetic apparatus from the adverse effects of stresses (He et al., 2002). The roles of PAs might therefore be the reduction of oxidative stress, maintenance of cellular pH, protection of pigment–protein complexes, protection of thylakoid membranes, regulation of chl biosynthesis and degradation pathways, and regulation of the enzymes related to those pathways. Supplementation of PAs relaxed salt stress effects and prevented chl degradation which restored and increased the contents of leaf chl (Li et al., 2014).

Inhibition of photosynthesis results in an over-reduction of the photosynthetic electron transport chain reactions, and it redirects photon energy into processes that promote the production of ROS and oxidative damage under salt stress (Sheokand et al., 2008; El-Shabrawi et al., 2010; Gill and Tuteja, 2010; Shabala and Munns, 2012). Salt stress creates ionic toxicity which inactivates the vital enzymes involved in major physiological processes, disrupted photosystem activity and mitochondrial electron transport chain causing imbalance between production and scavenging ROS, thereby amplify oxidative stress. Salt induced physiological drought, osmotic stress and stomatal closure are also responsible for ROS overproduction (Shabala and Munns, 2012). Mung bean plants exposed to salt stress showed higher accumulations of ROS, including H_2_O_2_ and $O_{2}^{•-}$, and showed higher LOX activity, which resulted in cellular oxidative damage, as reflected in increases in membrane lipid peroxidation or MDA levels (**Figures 2C–F**). Visual identification of H_2_O_2_ and $O_{2}^{•-}$, detected by histochemical staining (with DAB and NBT, respectively), also reflected a similar pattern of ROS generation under salt stress (**Figures 2A,B**). Exogenous application of PAs reduced LOX activity, contents and spots and of H_2_O_2_ and $O_{2}^{•-}$, and lipid peroxidation in mung bean seedlings (**Figures 2A–F**). In their study, Ikbal et al. (2014) reported that PAs biosynthesis was correlated with reduction spots of H_2_O_2_ and $O_{2}^{•-}$ in grapevine leaves. Reductions in LOX activity and reduced MDA contents under salt stress were also observed in previous studies (Nahar et al., 2015). PAs act as ROS and free-radical scavengers (Gill and Tuteja, 2010), enhance antioxidant machinery (Gill and Tuteja, 2010; Kubiś et al., 2014), PAs act as molecular chaperones and PAs bind to negatively charged surfaces and protect membranes and biomolecules (Kusano et al., 2008). Exogenous Put had a protective effect on salt-induced membrane damage in chick pea, where increased activities of antioxidant enzymes decreased ROS generation (Sheokand et al., 2008). Similar results were reported in grapevine plants where PAs biosynthesis increased significantly (Ikbal et al., 2014).

A balanced state of cellular ROS equilibrium is maintained by the interaction between enzymatic and non-enzymatic antioxidants which combat oxidative damage. Constitutes the first line enzymatic defense against ROS, SOD converts $O_{2}^{•-}$ to H_2_O_2_, and H_2_O_2_ is readily detoxified by CAT by through conversion of H_2_O_2_ to H_2_O and O_2_ (Gill and Tuteja, 2010). Mung bean seedlings showed increased SOD and decreased CAT activity, acutely increased $O_{2}^{•-}$ and H_2_O_2_ contents in salt-stressed seedlings, supported by previous studies (Nahar et al., 2016). PAs maintained same SOD and increased CAT activities (**Table 1**) in salt-stressed plants which overwhelmed overproduced $O_{2}^{•-}$ and H_2_O_2_ levels and improved leaf appearance by reducing damage spots (of $O_{2}^{•-}$ and H_2_O_2_) (**Figures 2A,B**; **Table 1**). APX, MDHAR, DHAR, and GR are the enzymes of the ascorbate-glutathione cycle which has major roles in ROS scavenging process and regenerating AsA and GSH. APX decomposes H_2_O_2_ via oxidation of AsA to DHA. Ascorbate reacts with $O_{2}^{•-}$, H_2_O_2_ to form MDHA or DHA. This reaction leads to DHA accumulation, which is harmful for plant cells. AsA is regenerated from DHA by MDHAR and DHAR, where NADPH and GSH are used as electron donors, respectively (Gill and Tuteja, 2010). Increased APX activity (**Table 1**) and DHA content (**Figure 3B**) and decreased AsA content (**Figure 3A**) decreased the AsA/DHA ratio (**Figure 3C**) of mung bean seedlings corroborating higher content of ROS. The increase in GSH level was not significant in salt-affected mung bean seedlings in the present study (compared to control), which showed increased ROS content and lipid peroxidation (**Figure 2**). Application of PAs increased activity of DHAR (**Table 1**), contents of AsA and AsA/DHA ratio, decreased DHA content (**Figures 3A–C**), increased the GR activity (enzyme involved in recycling of GSH) (**Table 1**), contents of GSH and GSH/GSSG ratio, decreased GSSG content (**Figures 3D–F**) which played major roles in the ROS (**Figures 2D–F**) detoxification process (Gill and Tuteja, 2010). Previous studies revealed the regulatory function of PAs in enhancing AsA-GSH cycle components and other antioxidant components that contributed to stress tolerance. Exogenous Spd application to salinized nutrient solution increased the antioxidant enzyme activities, including SOD, peroxidase (POD), and CAT, which alleviated salt-induced membrane damage and photosynthesis inhibition and promoted an increase in PAs content (Duan et al., 2008). Polyamine biosynthesis was correlated with enhanced APX, POD, SOD, and MDHAR activities, increased AsA content, decreased DHA levels, increased AsA/DHA ratio, increased GSH levels, decreased GSSG levels, and increased GSH/GSSG ratios in grapevine leaf tissues. Therefore, enhancement of PAs biosynthesis contributed to salt stress tolerance by upregulating ROS scavenging action and protecting the photosynthetic apparatus from oxidative damage (Ikbal et al., 2014). Using GSH as a substrate, GPX catalyzes the reduction of H_2_O_2_ and organic lipid hydroperoxides (Gill and Tuteja, 2010). GST conjugates GSH to a range of reactive aldehydes and xenobiotics to convert water-soluble and less toxic products. GSTs bear peroxidase activity, which reduces oxidative stress (Gill and Tuteja, 2010). The mung bean seedlings in this experiment showed a higher GPX and GST activity under salt stress, when compared to unstressed controls. GPX activity increased but GST activity did not increase further after PAs addition to salt-stressed plants, when compared to salt stress alone (**Table 1**). PAs have protective effects on proteins/antioxidant enzymes; PAs form complexes with SOD, GPX, and CAT for which these enzymes function more efficiently, compared with isolated enzymes (Li et al., 2014).

Methylglyoxal is a highly cytotoxic compound that causes protein and DNA degradation. MG disrupts the antioxidant machinery and act as mediator for $O_{2}^{•-}$ generation, causing an oxidative burst (Yadav et al., 2005). The mung bean seedlings had high MG content under salt stress. The glyoxalase system comprises of two enzymes, Gly I, and Gly II, which catalyze the detoxification reaction of MG to D-lactate. This detoxification process occurs mainly via two steps: Gly I converts MG to SLG utilizing GSH, while Gly II converts SLG to D-lactic acid, and regenerates GSH (Yadav et al., 2005). Exogenous Put, Spd, and Spm application enhanced Gly II activity and increased the contents of GSH, which reduced MG toxicity by reducing its content (**Figures 4A–C** and **5D**). Glutathione homeostasis and enhanced glyoxalase system activity were the biomarkers for salt tolerance in a Pokkali cultivar of rice (El-Shabrawi et al., 2010). Hasanuzzaman et al. (2011) reported that enhanced salt tolerance in wheat seedlings was partly contributed by increased activities of Gly I and Gly II. Modulation of PAs and MG in polyethylene glycol-affected white spruce was reported by Kong et al. (1998). The glyoxalase system was studied mostly in animals and microbes but not extensively in plants. It can be considered in stress specific studies.

The ratio of free [(Spd + Spm)/Put] is considered more important than the individual contents of Put, Spd, and Spm. A high [(Spd + Spm)/Put] ratio is crucial for imparting plant stress tolerance (Duan et al., 2008; Yang et al., 2010). Decreases in free Spd and Spm and increases in free Put decreased the [(Spd + Spm)/Put] ratio (**Figures 5A–D**), which indicates increased susceptibility of mung bean plants to NaCl toxicity (Yang et al., 2010). Therefore, the increase in Spd and Spm contents and elevation of the [(Spd + Spm)/Put] free ratio by exogenous application PAs with salt stress (**Figure 5D**) was critical in improving NaCl tolerance, in agreement with other published results showing that modulation of PAs was correlated with an altered physiology and biochemistry toward development of plant stress tolerance (Duan et al., 2008; Yang et al., 2010).

Higher NaCl concentration in the soil primarily causes ionic and osmotic stress that interrupts water transportation and reduces stomatal conductance, the efficiency of photosystems, RuBisCo activity, CO_2_ assimilation, and photosynthesis, thereby reducing growth in salt-affected plants (Shu et al., 2012). In the present study, the overall tolerance was estimated by seedling growth and development. Exogenous application of PAs to salt-affected mung bean seedlings resulted in better seedling growth, as determined by their higher plant height, root length, and DW, when compared to salt-affected seedlings (**Table 2**) which is supported by previous findings. Exogenous Spd positively affected the photosynthetic and xanthophyll cycle in salt affected cucumber seedlings. Spermidine alleviated the salt-mediated decline in photosynthetic efficiency by enhancing the maximum quantum efficiency and actual efficiency of photosystem II (PSII), and improving the net photosynthetic rate, which improved overall growth performance of cucumber seedlings (Shu et al., 2012). Polyamine biosynthesis was correlated with oxidative stress protection of photosynthetic apparatus, enhancement of PSII quantum yield in grapevine plants that improved photosynthesis and plant growth (Ikbal et al., 2014).

## Conclusion

**FIGURE 6:**
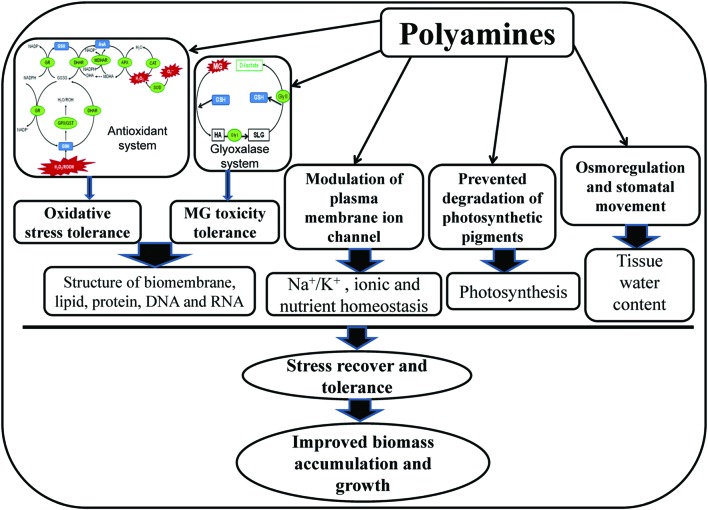
**Mechanism of PAs-induced salt stress tolerance.** In glyoxalase system, HA indicates hemithioacetal and SLG indicates S-D-lactoylglutathione.

In present study, salt stress created ionic and osmotic stress, disrupted biochemical processes, and altered PAs metabolism, resulted phytotoxicity in mung bean seedlings. High cellular Na content, imbalances in the mineral nutrients, oxidative damage, MG toxicity, and growth inhibition were the characteristic symptoms of salt stress affected mung bean seedlings. Polyamines played diversified roles in imparting salt stress tolerance in mung bean seedlings in the present study. The possible mechanism of PAs-induced salt stress tolerance has been presented in **Figure 6**. Exogenous PAs supplementation to salt-stressed mung bean plants modulated the endogenous levels of Put, Spd, and Spm and increased the (Spd+Spm)/Put ratio which might have regulatory roles in alteration of physiological features and stress defense mechanism of salt affected mung bean seedlings. Exogenous PAs application in mung bean seedlings increased content of AsA and AsA/DHA ratio, GSH content and GSH/GSSG ratio, enhanced activities of CAT, DHAR, GR, GPX which reduced ROS production, oxidative stress and subsequence membrane lipid peroxidation. PAs application in salt affected seedlings increased the content of GSH and activities of Gly I and Gly II enzymes which played vital roles in reducing MG toxicity. Reduction of ROS and MG by PAs were manifested through biomembrane and biomolecules protection and reduced oxidative damage in mung bean seedlings. Application of PAs prevented Na influx/toxicity and improved nutrient homeostasis in salt affected mung bean seedlings probably by regulating the plasma membrane ion channel. In salt affected mung bean seedlings, PAs increased Pro content which helped to increase leaf RWC, compared to salt stress alone. Osmoregulation and maintaining tissue water are vital for smooth running of physiological processes smoothly, stress recovery and tolerance which were imparted by PAs under salt stress. Preventing degradation, PAs improved potosynthetic pigment contents which might increase photosynthesis and growth of mung bean seedlings in present study.

## Author Contributions

KN, MH, and MF conceived and designed the experiments; KN, MA, AR, JM, and TS performed the experiments; MH analyzed the data; MF contributed reagents/materials/analysis tools; KN and MH wrote the manuscript. All authors read and approved the final manuscript.

## Conflict of Interest Statement

The authors declare that the research was conducted in the absence of any commercial or financial relationships that could be construed as a potential conflict of interest.

## Abbreviations

AO: ascorbate oxidase
APX: ascorbate peroxidase
AsA: ascorbic acid (ascorbate)
BSA: bovine serum albumin
CAT: catalase
CDNB: 1- chloro-2, 4-dinitrobenzene
chl: chlorophyll
DHA: dehydroascorbate
DHAR: dehydroascorbate reductase
DTNB: 5,5′-dithio-bis (2-nitrobenzoic acid)
EDTA: ethylenediaminetetraacetic acid
Gly I: glyoxalase I
Gly II: glyoxalase II
GR: glutathione reductase
GSH: reduced glutathione
GSSG: oxidized glutathione
GPX: glutathione peroxidase
GST: glutathione *S*-transferase
LOX: Lipoxygenase
MDA: malondialdehyde
MDHA: monodehydroascorbate
MDHAR: monodehydroascorbate reductase
MG: methylglyoxal
NADPH: nicotinamide adenine dinucleotide phosphate
NTB: 2-nitro-5-thiobenzoic acid
Pro: Proline
ROS: reactive oxygen species
RWC: relative water content
SLG: *S*-D-lactoylglutathione
SOD: superoxide dismutase
TBA: thiobarbituric acid
TCA: trichloroacetic acid
